# Dietary fiber pectin: challenges and potential anti-inflammatory benefits for preterms and newborns

**DOI:** 10.3389/fnut.2023.1286138

**Published:** 2024-01-12

**Authors:** Janaina L. S. Donadio, João Paulo Fabi, Marcelo B. Sztein, Rosângela Salerno-Gonçalves

**Affiliations:** ^1^Center for Vaccine Development and Global Health, University of Maryland School of Medicine, Baltimore, MD, United States; ^2^Department of Food Science and Experimental Nutrition, School of Pharmaceutical Sciences, University of São Paulo, São Paulo, Brazil; ^3^Food Research Center (FoRC), CEPID-FAPESP (Research, Innovation and Dissemination Centers, São Paulo Research Foundation), São Paulo, Brazil

**Keywords:** pectin, inflammation, intestine, newborn, preterm

## Abstract

Pectins, a class of dietary fibers abundant in vegetables and fruits, have drawn considerable interest due to their potential anti-inflammatory properties. Numerous studies have indicated that incorporating pectins into infant formula could be a safe strategy for alleviating infant regurgitation and diarrhea. Moreover, pectins have been shown to modulate cytokine production, macrophage activity, and NF-kB expression, all contributing to their anti-inflammatory effects. Despite this promising evidence, the exact mechanisms through which pectins exert these functions and how their structural characteristics influence these processes remain largely unexplored. This knowledge is particularly significant in the context of gut inflammation in developing preterm babies, a critical aspect of necrotizing enterocolitis (NEC), and in children and adults dealing with inflammatory bowel disease (IBD). Our mini review aims to provide an up-to-date compilation of relevant research on the effects of pectin on gut immune responses, specifically focusing on preterms and newborns. By shedding light on the underlying mechanisms and implications of pectin-mediated anti-inflammatory properties, this review seeks to advance our knowledge in this area and pave the way for future research and potential therapeutic interventions.

## 1 Chemical structures of pectins and general effects

Pectins, a class of dietary fibers naturally found in fruits and vegetables (1), exhibit highly viscous and water-soluble properties and are susceptible to fermentation by the intestinal microbiota (2). Multiple studies have suggested that incorporating pectins into infant formula is safe and could alleviate infant regurgitation and diarrhea, while also promoting overall physical development (3–10). Several clinical trials involving regurgitating infants fed with a pectin-thickened formula have reported reduced regurgitation episodes (3–7). Furthermore, due to its remarkable water-retention properties (11), pectin may also influence infant stool frequency, consistency, and potentially reduce the incidence of diarrhea (3, 8, 9, 12). Pectins, being extracted from plant material without undergoing chemical modification, primarily consist of polysaccharides with a small proportion of oligosaccharides (13). While there are structural differences between pectin oligosaccharides (POS) and human milk oligosaccharides (HMOs) (14), the ingestion of POS has been shown to influence the composition of the infant’s fecal microbiota and provide benefits akin to HMOs, including a decreased risk of infection by pathogenic bacteria and virus (15, 16). It is worth noting that HMOs represent the most significant solid component in breast milk, surpassing carbohydrates and fat (17). HMO concentration is highest in colostrum (20 g/L) and then decreases by about 20% at day 30 of lactation (18).

**Figure 1:**
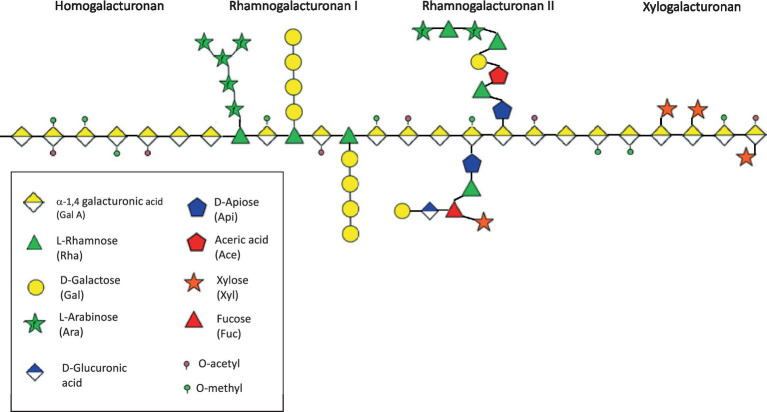
Schematic structure of pectins.

The molecular structure of pectins is notably complex, consisting of a homogalacturonan (HG) backbone core region comprising 1,4-α-D-galacturonic acid (GalA) units that can undergo methylation or acetylation, along with branched regions primarily composed of rhamnogalacturonan type I (RGI) (19). The HG backbone incorporates monosaccharides with β-(1,3)-D-xylose residues (xylogalacturonan) or branched structures with alternating α-(1,4)-GalA and α-(1,2)-rhamnose (Rha), the latter of which can be linked to RGI (20). RGI possesses intricate side chains of neutral sugars attached to the rhamnose residues, while the HG backbone may also include complex branches like aceric acid and apiose with distinct side chains, referred to as rhamnogalacturonan type II (RGII) (21). After HG, RGI is plants’ second major pectic fraction (21) (Figure 1). While pectins share the same fundamental repeating elements, their quantities and chemical structures can vary depending on their source, location within the plant, and the extraction methods employed (15). Pectins demonstrated varying degrees of esterification and a wide range of molecular weights, ranging from 60 to 900 kDa, precluding their intestinal absorption (19, 20, 22, 23). Notably, the structural characteristics of pectin directly influence the development of gut microbial communities (15).

In addition, pectins can have dual immunomodulatory effects: (a) direct interactions with the intestinal barrier and engagement with immune receptors, such as Toll-like receptors (TLRs), resulting in reduced inflammation, and (b) indirect effects via modulation of the gut microbiota through fermentation and production of SCFA (24–26). These properties are particularly relevant in intestinal inflammatory diseases, such as necrotizing enterocolitis (NEC), which remains a leading cause of mortality in premature neonates (27). In this review, we aim to provide a comprehensive overview of the immunomodulatory effects of pectins in the context of preterms and newborns and their potential role in modulating the neonatal gut epithelial barrier and microbiota. Understanding the impact of pectins on the epithelium and microbiota can offer valuable insights into their potential therapeutic applications for mitigating intestinal inflammation and promoting neonatal health.

## 2 Preterm microbiota

Preterm neonates exhibit a distinct gut microbiota composition compared to full-term neonates (28). Unlike vaginally born infants, preterm neonates delivered by cesarean section (C-section) tend to be colonized by maternal skin bacteria, leading to an abundance of *Staphylococcus*, *Corynebacterium*, *Propionibacterium* spp., and a deficiency of *Lactobacillus*, *Bifidobacterium*, and *Bacteroides* (28). This difference in microbiota colonization can significantly affect the neonate’s immune system (28, 29). Notably, C-section delivery has been associated with an increased risk of celiac disease, asthma, obesity, and type 1 diabetes in newborns (29). During vaginal delivery, *Escherichia coli*, *Staphylococcus*, and *Streptococcus*, play a crucial role in creating an anaerobic environment, which allows the colonization of strict commensal anaerobes such as *Bacteroides, Clostridium,* and *Bifidobacterium* spp. (29, 30). The diversity of the infant gut microbiota continues to increase over time with a significant shift at weaning (31, 32). This process is delayed in preterm neonates delivered by C-section, potentially affecting the growth of commensal anaerobic bacteria (29).

Apart from the mode of delivery, other factors can disrupt the neonate’s microbiota, including early antibiotic use and feeding practices. Early antibiotic administration reduces microbiome diversity in the neonate’s stool and eliminates the growth of commensal bacteria impairing the innate immune defense (29, 33, 34). Microbial colonization triggers and accompanies rapid morphological and functional changes in the gut (31, 32). It has been proposed that impaired colonization in preterm infants leads to dysmotility of the intestinal tract, and uncontrolled inflammation, triggering disease states including neonatal NEC (27, 35). The host’s central strategy to maintain its homeostatic relationship with the microbiota is to minimize contact between the microbiota and the epithelial cell surface, thereby limiting tissue inflammation (36). This segregation is partially accomplished by secretory-IgA (SIgA) (37). However, the presence of SIgA, a predominant immunoglobulin in the human gut, relies on the gastrointestinal tract’s colonization by microbiota. SIgA is critical in attenuating inflammatory reactions in the intestine, particularly in preterm infants (37–40). For example, germ-free mice, lacking microbiota, demonstrate a marked reduction of SIgA in the gut (41–43). Moreover, previous studies have shown that pectin-fed animals have significantly higher levels of SIgA and IgA than controls (e.g., cellulose-fed animals) (44–47).

The feeding modality can also shape the infant’s gut microbiota. Formula feeding, for instance, can impact the microbiota by increasing the prevalence of *Clostridium difficile*, *Bacteroides fragilis*, and *Escherichia coli*, while reducing the prevalence of beneficial commensal bifidobacteria (29). Lack of breastfeeding prevents neonates from acquiring prebiotics from human breast milk, resulting in lower microbial diversity and unusual gut colonization with pathogenic proteobacteria, such as *Enterobacter, Escherichia,* and *Klebsiella* in preterm newborns (48–50). The immature intestinal mucosal barrier in preterms also allows the passage of pathogenic bacteria and bacterial toxins through the epithelial cells (49). Finally, infants can inherit bacteria associated with antibiotic resistance from their mothers through breastfeeding (51).

Since, pectin and POS are fermented in the intestine by *Bacteroides, Bifidobacteria*, *Lactobacilli*, Enterococcus, and *Clostridium* (13), it is anticipated that the microbial community in preterm neonates will differ in its ability to metabolize pectin substrates and, consequently, produce short-chain fatty acids (SCFA) compared to full-term babies. Unlike full-term babies, preterm infants with a deficiency of *Lactobacillus*, *Bifidobacterium*, and *Bacteroides* are expected to exhibit a reduced capacity for pectin degradation. *Bacteroides*, known for their pivotal role in breaking down various plant polymers, possess many carbohydrate-active enzyme (CAZymes) genes (52). Furthermore, pectin substrates may also foster the growth of beneficial bacteria. For instance, RGI-enriched citrus pectin has been shown to selectively promote the growth of *Bifidobacterium*, *Lactobacillus*, and *Faecalibaculum* spp. (53). Larsen and colleagues have also demonstrated that the abundance of beneficial bacteria such as *Bifidobacterium*, *Christensenellaceae*, *Prevotella copri*, and *Bacteroides* spp. can either increase or decrease depending on the specific pectin substrate, suggesting that the microbial community in preterm infants can be modulated using structurally different pectins to promote the growth of more beneficial bacteria (54).

## 3 Pectins and intestinal barrier in NEC

NEC is a severe inflammatory disease of premature neonates’ gastrointestinal tract, characterized by intense intestinal necrosis (55, 56). NEC’s mortality rate can reach 30%, leaving the survivors with severe neurodevelopmental delays (56, 57). While the exact mechanisms responsible for NEC development are still debated, they may involve factors such as the prematurity of the intestine, intense production of inflammatory cytokines, defective mucus production, and low expression of tight junctions (TJ) proteins, leading to increased intestinal permeability and penetration of pathogenic bacteria and toxin, causing tissue injury and intestinal necrosis (56, 58). One potential mechanism by which pectins can contribute to preventing intestinal inflammatory diseases is by preserving the integrity of the intestinal layer and enhancing mucosal immunity (24). The intestinal layer is safeguarded by several physical barriers, including gastric acid, the mucus layer, and a tight monolayer of intestinal epithelial cells (IECs) held together by TJ to prevent the transfer of pathogens and toxins from the lumen into the circulation (59). These TJ play a crucial role in maintaining the gastrointestinal barrier’s integrity by regulating the permeability of the intestinal cell layer (58).

The mucus layer is a protective barrier separating the IECs from the luminal content and the microbiota. It mainly consists of mucins, glycoproteins produced by goblet cells, with MUC2 being the predominant mucin in the small and large intestines (59). Pectins can stimulate the production of MUC2 (60). Additionally, the mucus layer contains defensins, antimicrobial components derived from Paneth cells, and SIgA that protects against pathogen invasion (61–63). The mucus coating is composed of two layers: an outer layer, housing commensal bacteria, and an inner layer, which acts as a barrier against bacterial penetration (64). Neonates with NEC may have fewer goblet cells producing mucus, resulting in impaired mucus production after infection (65, 66). While not yet confirmed in humans, a neonatal rat NEC model demonstrates that HMOs provide protection against NEC (67). Survival rates and pathology scores show significant improvement when HMOs are introduced into orally administered formula. These beneficial effects are hypothesized to be mediated through specific receptors that mimic pathogen lectins, preventing interactions with host glycans. It is worth noting that pectin and POS also exhibit antiadhesive and antimicrobial properties, similar to HMOs (14). Additionally, pectins possess mucoadhesive properties by adhering to densely mucin-grafted glycans (68) and preventing pathogen colonization.

NEC patients often exhibit higher production of pro-inflammatory cytokines, such as TNF-α and IL-1β, which increase intestinal TJ permeability, causing bacterial translocation and boosting the inflammatory state in the gut (58). Pectins can also directly interact with TLR signaling pathways, thereby reducing inflammation (69, 70). For instance, pectins have been found to inhibit IL-6 secretion induced by TLR2-1 (70, 71). Additionally, TLR4 can serve as a receptor for non-canonical ligands, including carbohydrates present in pectins (72). It is believed that the increased expression of TLR4 on IECs might explain the excessive inflammatory response with high production of pro-inflammatory cytokines including IL-6, IL-8, and TNF-α found in NEC patients (73). TLR4 is responsible for detecting lipopolysaccharide (LPS), a critical outer membrane component of gram-negative bacteria, which stimulates the NF-κb pathway to produce pro-inflammatory cytokines, such as IL-8 and TNF-α (74). TLR4 also increases intestinal stem cell apoptosis and decreases IEC proliferation and migration, impairing intestinal mucosal healing and regeneration, thereby favoring NEC development (56).

## 4 Impact of pectin structure and origin on its function

Several studies have observed that pectins with different structures have distinct modulatory effects on the immune system. Most of the studies were conducted using pectins from citrus (e.g., lemon) (75, 76), but also with pectins from apple (77), cacao (78), and papaya (69). Pectins have a backbone composed of GalA that varies in the degree of methyl-esterification (DM), and the immunomodulatory effects of pectins are dependent on the DM. *In vitro* and *in vivo* studies (Tables 1, 2, respectively) showed that pectins, with different chemical structures and degrees of DM, can strengthen the mucus layer by directly stimulating mucin production by goblet cells.

**Table 1:** *In vitro* studies on the immunoregulatory activities of pectins.

Dietary source	Specific pectin characteristic	*In vitro* model	Immunomodulatory activity of pectins	References
Apple	Apple pectin (AP)	Colon cancer cell lines HT29	AP: ↓ TLR4 expression in the cell membrane	Liu et al. (77)
			Redistributed TLR4 to the cytoplasm	
			AP+LPS: ↓ TNF-a production	
Cacao pod husk	Modified with different degrees of DM and DE	Murine peritoneal macrophages	MOP: ↑ anti -inflammatory IL-10	Amorim et al. (78)
	OP (native pectin), MOP (modified pectin)		↑ pro-inflammatory TNF-a and IL-12 production in macrophages	
Citrus	Pectins with different DE (DE30, DE60, DE90)	LPS-activated macrophages RAW264.7 and murine peritoneal macrophages	All pectins: ↓ iNOS and COX-2 expression	Chen et al. (79)
			DE90: ↓ NF-kB activation	
Citrus	Pectins with different DE (DE30, 60, 90)	PBMC	DE60, DE90: ↓ IL-1b and ↑ IL-1ra in a dose-dependent manner	Salman et al. (80)
			DE30: ↓ IL-10 in a dose-dependent manner	
Citrus	Native citrus and orange pectins	Murine macrophage RAW264.7	Orange pectin ↓ IL-6	Ishisono et al. (76)
Orange				
Lemon	Pectins with all DM already in low doses	HEK-Blue hTLR4 reporter cell line	↑ TLR4	Vogt et al. (81)
	Pectins with high and low DM (DM30 and DM74)	HEK-Blue hTLR2 reporter cell line	DM74: ↑ TLR2 in a dose-dependent manner	
			DM30 and DM74: Improved epithelial barrier integrity in intestinal epithelial cells	
Lemon	Pectins with high and low DM	HEK-Blue hTLR2 reporter cell line	DM7: ↓ TLR2	Sahasrabudhe et al. (70)
			DM7 and DM75: ↓ IL-6 and IL-10 secretion in human dendritic cells	
			DM7: ↓ IL-6 secretion in mouse macrophages RAW264.7	
Lemon	Citrus pectin (CP) with non-esterified Gal-A residues	HEK-Blue mTLR2 reporter cell line	All pectins ↓ TLR2 in a dose-dependent manner	Beukema et al. (82)
			Pectins with a blockwise distribution of non-esterified Gal-A have a stronger effect on mTLR2 inhibition	
Lemon	lemon pectins with high and low DM	HEK-Blue hTLR2 reporter cell line	DM33: ↓ TLR2-1	Beukema et al. (71)
			DM52: ↓ TLR2-1, TLR3, TLR4	
			All pectins: ↓ IL-6	
Orange	orange pectins with high and low DM	HEK-Blue hTLR2, 3, 5, 8, 9 reporter cell lines	DM32: ↓ TLR3 and TLR8	
			DM64: ↑ TLR2	
			DM64: ↓ TLR2-1, 3, TLR5, TLR8, TLR9	
MCP	Pectasol-C	Human lymphocytes	↑ T-cytotoxic, B and NK cells in a dose-dependent manner	Ramachandran et al. (75)
		K562 chronic myeloid leukemia cells	↑ NK-cell activity on K562 cells in a dose-dependent manner	
Papaya	Unripe pectin (longer chain and low DM)	HEK-Blue hTLR3,4 and 9 reporter cell lines	↓ TLR3 and TLR9	Prado et al. (69)
			All pectins: ↑ TLR2 and TLR4	

PBMC, human peripheral blood mononuclear cells; DE, degree of esterification; DM, degree of methyl-esterification; TLR, toll-like receptor, MCP, modified citrus pectin.

**Table 2:** *In vivo* studies on the immunoregulatory effects of pectins.

Pectin source	Chemical structure	*In vivo* model	Immunomodulatory activity of pectins	References
Apple	Oligogalactan from apple	5-week old ♂ mice of Institute of Cancer Research (20/group) DMH/DSS-induced mouse colitis	↓ tumor formation in the colon	Liu et al. (77)
			↓ inflammation in the colon	
			↓ TLR4 expression in colonic tissue	
			↓ TNF-a	
Apple	Native apple pectin (AP)	4-week old ♀ Balb-c mice (6/group) DSS- induced mouse colitis	AP: ↑ fecal and mesenteric lymph node (MLN) IgA	Lim et al. (44)
			DS+AP: ↑ MLN IgA	
			AP: ↓ CD4+/CD8+ cells	
Apple	Native	6-weeks old ♂ BALB/c mice and IL-10^-/-^ mice (5/group)	↓ TNF-a production	Ye and Lim (83)
			↓ IgM and IgG expression in spleen in IL-10^−/−^ mice	
			↑ CD4^+^ and CD8^+^	
Artichoke and citrus	Artichoke pectin (AP) Modified Artichoke pectin (APwA) Citrus pectin (CP)	7-9-weeks old ♂ C57BL/6 mice (6/group) DSS- induced mouse colitis	AP and APwA: ↓ TNF-a, iNOS, ICAM-I expression	Sabater et al. (84)
			AP and CP: ↓ IL-6 production	
			AP: ↑ MUC-1 and occluding expression	
			CP: ↑ ZO-1 and villin expression	
Citrus	Citrus pectin and MCP (Pectasol-C)	8-week old ♀ BALB/c mice (5/group)	Both pectins: ↑ IL-4, IL-17 and IFN-g production	Merheb et al. (85)
			MCP: ↑ TNF-a production	
			CP: ↓ IL-1b production	
Lemon	Lemon pectins with DM7	7-10-weeks old ♀ C57BL/6 mice (n/group n.i.) Doxorubicin-induced ileitis	↓ TNF-a and IL-6 production	Sahasrabudhe et al. (70)
			↓ neutrophil influx	
			↓ apoptosis in the crypts	
Lemon	Pectins with low DM (DM18) and high Gal-A residues	10-weeks old ♀ C57BL/6 mice (n/group n.i.) Doxorubicin-induced ileitis	↓ apoptotic cells in the crypts	Beukema et al. (82)
			↓ histopathological score	
			Prevented doxorubicin-induced villus degeneration	
			All pectins: ↓ neutrophil influx	
			All pectins: ↓ IL-6 and MCP-1 secretion in the peritoneal cavity	
Lemon	Low DM pectins (DM7)	♀ BALB/c mice (6-8/group) Caerulein-induced acute pancreatitis	↓ IL-6, IL-1b and TNF-a production	Sun et al. (86)
			Improved epithelial barrier integrity	
			↑ occludin expression	
MCP	MCP Pectasol-C	Weeks old ♂ Wistar rats (15/group)	↓ TLR4, MyD88, pNF-kB-p65 expression	Xu et al. (87)
			↓ IL-1b, IL-18, TNF-a	
Orange	Pectins with DM64	10-weeks old ♀ C57BL/6 mice (5/group) Citrobacter rodentium-induced colitis	Prevented intestinal barrier dysfunction	Beukema et al. (26)
			Enhanced microbiota diversity	
Citrus	Native citrus pectin	7-9-weeks old ♂ C57BL/6 mice (n/group n.i.) DSS-induced colitis	↓ IL-1b, TNF-a, IL-17A in the colon	Ishisono et al. (76)
Orange	Orange pectin: more arabinose and galactose, high content of neutral sugar side chain	TNBS-induced colitis	Ameliorates TNBS-induced colitis in a side chain-dependent manner	
Pear	Native Asian pear pectin	6–8-week ♂ BALB/c mice (11/group)	↓ IFN-g and ↑ IL-5 in bronchial fluid	Lee et al. (88)
			↑ IFN-g and ↓ IL-5 in splenic cells	
			normalized pulmonary histopathological changes	
			↓ serum IgE	

DE, degree of esterification; DM, degree of methyl-esterification; DMH, 1,2-dimethylhydrazine; DSS, dextran sulfate sodium; MCP, modified citrus pectin; TNBS, 2,4,6-trinitrobenzoic sulfonic acid; n/group n.i.: number per group not informed; ♂: male; ♀: female; ↓: reduced secretion/expression; ↑: increased secretion/expression.

*In vitro* studies have shown that pectins from citrus, lemon, and orange with higher DM (DM52 to 90) were able to reduce expression of NF-κB, IL-1β, IL-6, and IL-10 in a dose-dependent manner, reduce the activation of TLR2-1, TLR3, and TLR4, and increase the activation of TLR2 (70, 71, 79–81). In contrast, pectins with low DM (DM7 to 30) from citrus, lemon, orange, and papaya improved epithelial barrier integrity, reduced secretion of IL-10, and IL-6 in a dose-dependent manner, and reduced the activation of TLR2, TLR2-1, TLR3, TLR8, TLR9 [69; 70; 71; 80; 81]. Interestingly, the reduction of IL-6, iNOS, and COX-2 expression, the activation of TLR2 and TLR4, and the inhibition of TLR2 were achieved for some pectins regardless of the DM (69, 71, 79, 81, 82). Pectins with a low DM of 50 can penetrate the mucin layer and interact with IEC (24). Treating polarized monolayers of human T84 intestinal epithelial cells with lemon pectin, especially pectins with DM30 and DM74, can also improve their transepithelial electrical resistance (81). Moreover, low-methoxyl pectin from lemon can restore epithelial barrier integrity by increasing TJ protein expression such as occluding and zonula occludens (ZO-1) (86). The immunomodulatory effects of pectins in *in vitro* studies has been summarized in Table 1.

An increasing amount of evidence on *in vivo* models (Table 2) suggests that pectins from apple, artichoke, citrus, lemon, orange, and pear can have anti-inflammatory properties, including the capacity to regulate cytokine production, macrophage activity, and TLR expression (83, 85, 87, 88). Apple pectin treatment decreased the production of TNF-α and inflammation in the colon in a DMH/ DSS-induced colitis model (77). Native artichoke pectin and modified artichoke pectin reduced the expression of IL-6, TNF-α, iNOS, and ICAM and also increased the expression of TJ proteins MUC1 and occludin (84). Citrus and lemon pectins with low DM reduced the production of IL-1β, IL-6, and TNF-α, reduced neutrophil migration, improved epithelial barrier integrity, and increased expression of occludin in doxorubicin-induced ileitis (70, 82) and carerulein-induced pancreatitis (86). A compilation of the immunomodulatory effects of different pectins in animal models is shown in Table 2.

It is essential to acknowledge that many referenced *in vivo* studies (Table 2) were conducted on mice or rats a few weeks old, not neonates. While *in vitro* experiments (Table 1) provide some insights into cell responses, the question remains whether preterm and neonatal cells will exhibit similar responses as the established cell lines. Hence, there is a strong rationale for developing advanced models, like neonatal gut organoids cultured under anaerobic conditions, which can accurately replicate the interactions between pectin, neonatal gut cells, and microbiota to validate and extend these findings.

## 5 Pectin consumption stimulates the production of SCFA by the microbiota

Pectin consumption stimulates the production of SCFA by the gut microbiota, which has beneficial effects on gut health (24, 25). SCFA, such as butyrate, stimulates mucin secretion *in vitro* through the upregulation of *MUC3*, *MUC4,* and *MUC12* genes in the LS174T human colorectal cancer cell line (89). A low-fiber diet causes a shift in the gut microbiota to mucin degraders bacteria (e.g., *A muciniphila, B. thetaiotaomicron*) to fulfill their energy requirements (90). When pectins and other soluble fibers like inulin and β-glucan are fermented in the large intestine, they produce SCFAs due to their higher viscosity and solubility. This fermentation process stimulates the growth of healthy bacteria and reduces the growth of pathogenic bacteria (91). SCFAs, particularly acetate, propionate, and butyrate, serve as a primary energy source for colonocytes and play a crucial role in maintaining normal colonic function (92). They help in lowering intestinal pH, stimulating electrolyte and fluid absorption, increasing blood flow, and preventing pathogen overgrowth and intestinal inflammation (1, 91, 93, 94).

The production of SCFAs also contributes to reducing inflammation through different mechanisms, including the activation of G-protein-coupled receptors (GPRs), which inactivate the NF-κB pathway in immune and intestinal cells (91, 95). GPR41 and GPR43 are important for immune surveillance in the colon, stimulating the secretion of cytokines IL-1β and IL-18 (91). GPR43 is mainly expressed in innate immune cells, such as neutrophils and macrophages (96). GPR109, activated by butyrate, inhibits the pro-inflammatory NF-κB pathway (91, 96). Furthermore, SCFAs inhibit the production of pro-inflammatory cytokines IL-8, IL-12, IL-1, and TNF-α, and decrease NF-κB expression (95). The main three GPRs activated by SCFA are expressed in the enteroendocrine cells of the colonic epithelium, the polymorphonuclear immune cells, and smooth muscle cells (91).

Butyrate, in particular, modulates immune cells, such as macrophages, dendritic cells, and lymphocytes, inhibiting the production of cytokines IL-12p70 and IL-23 (91). It also regulates the proliferation of stem cells from the intestinal crypts (97). SCFAs are crucial for regulating intestinal inflammation by controlling the migration of immune cells to sites of injury and modulating their activation state (95). Additionally, they inhibit histone deacetylases (HDACs) in the IECs and immune cells (92). HDCA inhibition reduces the expression of NF-κB in immune cells and, as a result, decreases the production of pro-inflammatory cytokines (92). Studies have shown that SCFA can reduce the pro-inflammatory response by modulating TLR4 signaling pathway, reducing leukocyte infiltration, increasing the production of the anti-inflammatory cytokine IL-10, and reducing the pro-inflammatory cytokines IL-6, IL-12, and TNF-α (92).

## 6 Conclusion and future directions

It is crucial to emphasize that neonates, unlike full-term infants, do not consume pectins through a regular diet. Nonetheless, there are two potential pathways through which pectins could inhibit NEC development. Firstly, direct immunomodulatory effects of pectins have been demonstrated both *in vitro* (Table 1) and *in vivo* studies (Table 2). Secondly, pectins may indirectly affect NEC by modulating the gut microbiota. Considering these pathways, pectins, which are natural compounds found in vegetables, could be added as a supplement to breast milk to help reduce inflammation in neonates with NEC or even mitigate the risk of NEC in preterm neonates within neonatal intensive care units.

Challenges and outstanding questions.

1. What are the best sources of pectin? Natural or “modified” pectins?

2. What is the minimum amount of pectin to observe anti-inflammatory effects?

3. Are the pectin anti-inflammatory effects age-related?

## Author contributions

JD: Funding acquisition, Writing – original draft, Visualization. JF: Supervision, Writing – review & editing. MS: Writing – review & editing, Funding acquisition. RS-G: Funding acquisition, Supervision, Writing – original draft.

## Figures and Tables

**Table:** 

DM	Methyl-esterification
GalA	Galacturonic acid
GPRs	G-protein-coupled receptors
HDACs	Histone deacetylases
HG	Homogalacturonan
HMOs	Human milk oligosaccharides
IBD	Inflammatory bowel disease
IECs	Intestinal epithelial cells
LPS	Lipopolysaccharide
NEC	Necrotizing enterocolitis
POS	Pectin oligosaccharides
RG	Rhamnogalacturonan
Rha	Rhamnose
SCFA	Short-chain fatty acids
TJ	Tight junctions
TLR	Toll-like receptor
